# A brief comment on liver resection for hepatocellular carcinoma

**DOI:** 10.1093/gastro/got019

**Published:** 2013-06-14

**Authors:** Haifeng Xu, Yilei Mao

**Affiliations:** Department of Liver Surgery, Peking Union Medical College Hospital, Peking Union Medical College & Chinese Academy of Medical Sciences, Beijing, China

Although only 30–40% of patients with hepatocellular carcinoma (HCC) are eligible for surgery, it remains the most feasible and efficient treatment [1, 2]. The three most important factors that have led to reduce mortality, with a 70% expectation of 5-year survival, are: i) better liver function assessment, ii) understanding of the segmental liver anatomy through more accurate imaging studies and iii) technical advances in surgical procedures [3]. Hepatic resection is the treatment of choice for HCC, especially in non-cirrhotic patients. Major resections can be carried out with low rates of life-threatening complications. Conversely, among patients who have cirrhosis, strict selection criteria are required to avoid treatment-related complications.

## Partial liver resection

In an HCC patient, both tumor size and degrees of histological changes of the underlying parenchyma will considerably influence the indication and the extent of partial liver resection. HCC tumors in patients with normal livers are often large (>10 cm) without vascular invasion and diagnosed when tumors are symptomatic. The only curative treatment is major hepatectomy with lymph node dissection, which is often well tolerated in the absence of underlying liver disease and with good regenerative capacity of the remnant liver. The long-term results of resection of HCC without chronic liver disease are much better than in patients with cirrhosis, with reported 5-year disease-free survival rates as high as 60–65% [4]. Intrahepatic recurrence should be treated whenever possible by repeat resection ablation. Therefore, partial resection in a diseased parenchyma increases risk due to impaired liver regeneration, altered texture of liver parenchyma, portal hypertension and coagulation defects [5]. There is a close relationship between the extent of resection and postoperative risk, which limits the indication of resection in patients with altered liver function and those with large tumors [6]. Indeed, partial resection in patients with diseased liver must follow two contradictory objectives: i) to be curative with resection of the tumor vascular territories and ii) to preserve as much liver parenchyma volume as possible to prevent postoperative liver failure.

## Anatomical resection

The anatomical territory of HCC ranges from sub-segment to lobe, according to the size of the tumor. Intrahepatic metastasis of HCC along the portal vein and the presence of satellite nodules up to 2 cm in size are the basis of anatomical liver resection [7]. Indeed, anatomical resections according to the architecture of the portal vein have the potential to remove undetected cancerous foci (portal vein metastases and satellite nodules) disseminated from the primary gross tumor. Moreover, anatomical resections of small solitary HCC achieve a significantly better overall and disease-free survival rate than limited resections, without increasing postoperative risk [8, 9]. However, the benefit of segmental resection may only become apparent in tumors between 2 and 5 cm in size. Below this size range, the risk of dissemination is considered to be negligible, with efficacy equivalent to local ablative therapy whereas, beyond this size, the majority of patients will already have macroscopic vascular invasion or satellite nodules that will dictate a high incidence of recurrence [10]. In the case of central tumors with undefined vascular territory, some authors have found a lower recurrence rate and better survival with a 2 cm margin, compared with a 1 cm margin [11], whereas other authors have not found differences, categorizing margins as <1 or >1 cm [8]. In fact, a wide margin up to 2 cm is required. It is proven that hepatic resection for HCCs >10 cm in diameter without macroscopic venous invasion is a safe and effective option. Spontaneous rupture of HCC and extension to surrounding structures, such as the diaphragm, the stomach or the colonic flexure, does not represent a contra-indication for resection if negative resection margins are attained. The presence of two or three nodules can be subjected to oncological surgical resection [12]. In selected patients with multifocal tumors, partial resection can be associated with stabilization of contralateral liver nodules by chemo-embolization or radiofrequency ablation [13]. Patients with HCC and tumor thrombus in the vena cava or in the portal trunk tend to have a poorer prognosis [14]. A major vascular involvement is generally associated with large tumors, for which no treatment can be anticipated. Yet it has been shown that, in a selected group of patients with normal liver function and excellent general status, extensive liver resection, associated with removal of the vascular thrombus, could achieve favorable survival results [14]. According to our own experience, anatomical resection, such as right hemihepatectomy or right three lobectomy, is relatively safe in some strictly selected patients.

## Laparoscopic liver resection

Laparoscopic liver resection represents 10–20% of all current liver resections, at least in the USA [15, 16]. Major obstacles for laparoscopic liver resection include a long learning curve, the difficulty of achieving a wide resection margin and performing anatomical resections, as well as technical difficulties in mobilization and parenchymal transection with risk of massive bleeding. The anticipated advantages of laparoscopic liver resection are its less aggressive approach (incision with less pain, fewer pulmonary complications and early recovery), less peritoneal dissection, avoidance of collateral ligation, reduced bleeding, minimal ascites and decreased postoperative liver failure [15]. These postulated factors could expand indications of liver resection in some Child-Pugh class B patients [15]. Moreover, compared with open liver resection, the reduced number of postoperative adhesions after laparoscopic liver resection facilitates subsequent salvage liver transplantation, with decreased morbidity [17]. In China, the suggested liver resection with laparoscopy can currently be performed in segments II, III, IV, V and VI of the liver.

Liver resection is an established treatment for HCC owing to its minimal surgical mortality and improved survival rate. Treatment guidelines for HCC will facilitate decision-making by both patients and physicians at every clinical step. Physicians need to recommend treatment options and allow the patient to choose.
